# Diversity in randomized clinical trials for peripheral artery disease: a systematic review

**DOI:** 10.1186/s12939-024-02104-8

**Published:** 2024-02-13

**Authors:** Chandler Long, Abimbola O. Williams, Alysha M. McGovern, Caroline M. Jacobsen, Liesl M. Hargens, Sue Duval, Michael R. Jaff

**Affiliations:** 1 Duke Vascular and Endovascular Surgery, Duke University Medical Center, Duke University, Durham, NC 27707 USA; 2 Health Economics & Market Access, Boston Scientific, Marlborough, MA 01752 USA; 3 Division of Cardiology, Department of Medicine, University of Minnesota, Minneapolis, MN 55455 USA; 4 Peripheral Interventions, Boston Scientific, Maple Grove, MN 55133 USA

**Keywords:** Peripheral artery disease, Health disparities, Demographic representation, Systematic review, Enrollment strategies, Endovascular interventions, Clinical trials

## Abstract

**Background:**

Significant race and sex disparities exist in the prevalence, diagnosis, and outcomes of peripheral artery disease (PAD). However, clinical trials evaluating treatments for PAD often lack representative patient populations. This systematic review aims to summarize the demographic representation and enrollment strategies in clinical trials of lower-extremity endovascular interventions for PAD.

**Methods:**

Following the 2020 Preferred Reporting Items for Systematic Reviews and Meta-Analyses (PRISMA) guidelines, we searched multiple sources (Medline, EMBASE, Cochrane, Clinicaltrials.gov, WHO clinical trial registry) for randomized controlled trials (RCTs), RCT protocols, and peer-reviewed journal publications of RCTs conducted between January 2012 and December 2022. Descriptive analysis was used to summarize trial characteristics, publication or study protocol characteristics, and the reporting of demographic characteristics. Meta-regression was used to explore associations between demographic characteristics and certain trial characteristics.

**Results:**

A total of 2,374 records were identified. Of these, 59 met the inclusion criteria, consisting of 35 trials, 14 publications, and 10 protocols. Information regarding demographic representation was frequently missing. While all 14 trial publications reported age and sex, only 4 reported race/ethnicity, and none reported socioeconomic or marital status. Additionally, only 4 publications reported clinical outcomes by demographic characteristics. Meta-regression analysis revealed that 6% more women were enrolled in non-European trials (36%) than in European trials (30%).

**Conclusions:**

The findings of this review highlight potential issues that may compromise the reliability and external validity of study findings in lower-extremity PAD RCTs when applied to the real-world population. Addressing these issues is crucial to enhance the generalizability and impact of clinical trial results in the field of PAD, ultimately leading to improved clinical outcomes for patients in underrepresented populations.

**Registration:**

The systematic review methodology was published in the International Prospective Register of Systematic Reviews (PROSPERO: CRD42022378304).

**Supplementary Information:**

The online version contains supplementary material available at 10.1186/s12939-024-02104-8.

## Background

Peripheral artery disease (PAD) is associated with serious adverse medical events and substantial healthcare spending [1, 2]. Significant disparities exist in the prevalence, diagnosis, and outcomes of PAD based on race and sex. While limited data comparing racial and ethnic differences in PAD prevalence is available [3], prevalence rates vary by geographic regions globally [4]. PAD prevalence in the United States (US) is higher among Black patients [3, 5] who also experience worse outcomes [6]. Additionally, Black, Hispanic, and Native American patients in the US are more likely to undergo amputations as a result of PAD [7–10], while individuals of Asian or Pacific Islander race experience a higher mortality burden when hospitalized for PAD [9].

Disparities by sex are evident as well. Global PAD prevalence is higher in women than in men [4]. In the US, women with PAD present at an older age and with more severe disease, and female sex is associated with more advanced PAD-related disability. However, women are also less likely to receive optimal medical therapy (i.e., statins) or surgical intervention than their male counterparts [11–14]. Notably, short-term complications after interventions [11] and above-the-knee amputations are more prevalent among women than men [14, 15]. Among US women with PAD, Black and Native American women experience higher mortality than White and Hispanic women [14].

In addition to substantial morbidity, PAD imposes a significant financial burden on patients and society. In the US, the direct medical costs of PAD amount to $6.3 billion [16]. Disparities in PAD diagnosis and treatment extend to differences in costs and utilization: among hospitalized patients with PAD, costs and length of stay differ significantly based on a patient’s race/ethnicity [9].

Endovascular interventions for PAD have shown promise in clinical trials [17, 18], but these trials often lack diverse patient groups that accurately represent the affected population [19–21]. Disparities in PAD care and the need to enhance diversity in clinical trials have been noted in previous studies [11, 22, 23], and multiple calls to address this lack of diversity exist [12, 24]. Therefore, this study seeks to identify and summarize the demographic representation and enrollment strategies employed in clinical trials of lower-extremity endovascular interventions for PAD. This review includes trials of patients with PAD undergoing lower-extremity endovascular interventions, specifically targeting the superficial femoral artery (SFA), femoropopliteal artery (FPA), popliteal artery, and tibial artery.

## Methods

This review followed the 2020 Preferred Reporting Items for Systematic Reviews and Meta-Analyses (PRISMA) guidelines. The systematic review methodology was published in the International Prospective Register of Systematic Reviews (PROSPERO: CRD42022378304) and Long et al. (2023) [25].

### Data sources and searches

Several sources were searched, including ClinicalTrials.gov, MEDLINE via OVID, EMBASE via OVID, Cochrane Controlled Register of Trials (CENTRAL), National Institutes of Health grants, and the World Health Organization International Clinical Trials Registry Platform (WHO ICTRP), which was accessed through Dr.Evidence™ (Santa Monica, CA) [26–28]. Additionally, Google Scholar was searched for protocols or publications that may not have been indexed in the trial registry. Manual searches of references of eligible publications were also performed. A comprehensive overview of the search strategy used in this study is available as a supplementary file (see Supplementary File, Table 1).

### Eligibility criteria

This review included any randomized controlled trials (RCTs) with a parallel group design that compared clinical outcomes of lower-extremity endovascular interventions, including patency rate, target lesion revascularization (TLR), all-cause mortality, amputation rates, amputation-free survival, minor or major amputations, serious adverse events/major adverse limb events (MALEs), change in ankle-brachial index, or improvement in Rutherford category. The inclusion criteria for RCTs in this review were: a sample size greater than 50 patients; published in English between January 2012 and December 2022; and inclusion of 12-month outcome data. Studies were excluded if they did not report the clinical outcomes of interest, if they reported the clinical outcomes of interest outside the 12-month period, or lacked a clinical trial registration number. Non-controlled studies, including those with a single-group assignment, single-arm design, or pragmatic study design, were excluded. The full list of eligibility criteria has also been published in Long et al., 2023 [25]. The search terms were applied following the population, interventions, comparators, outcomes, and setting (PICOS) framework, as detailed in Table 1.

**Table 1:** Study PICOS framework

P	Adults (≥ 18 years) diagnosed with PAD, critical limb ischemia, intermittent claudication, severe limb ischemia, or chronic limb-threatening ischemia
I	LE endovascular interventions for femoral/popliteal/tibial (percutaneous transluminal angioplasty (PTA), drug-eluting stent (DES), drug-coated balloon (DCB), and bare-metal stent (BMS)) in one treatment arm
C	LE endovascular interventions for femoral/popliteal/tibial (PTA, DES, DCB, and BMS)
O	i. Primary outcomes: Eligibility criteria of patients (inclusion and exclusion criteria); baseline demographic characteristics of patients enrolled and excluded (age, race/ethnicity, sex, etc.); and baseline clinical characteristics of patients enrolled and excluded (intermittent claudication, critical limb ischemia, Rutherford classification, diabetes, etc.)​ ii. Secondary outcomes: Reporting of outcomes by demographic characteristics (sex, race, etc.); enrollment/recruitment strategies (adaptive and targeted such as online, community, academic, etc.), participant facing-materials (availability of materials in other languages, including consent processes), diversification of trial investigators, trial protocols (inclusion of patient-centered processes), and patient reimbursement.​
S	Global (all countries)

*PICOS* Population, Interventions, Comparators, Outcomes, and Setting

### Data extraction, risk of bias, and statistical analysis

Title and abstract screening, as well as full-text screening, were performed independently by two reviewers (CMJ and AMM). Disagreements regarding the eligibility of the studies were resolved by a third reviewer (AOW). Data extraction was conducted using a data extraction form specifically developed for this review. Two reviewers (CMJ and AMM) performed data extraction, and a third reviewer verified the data for quality assurance and resolved any discrepancies or inaccuracies (AOW). Two independent reviewers (AOW and CMJ) evaluated the methodological quality of eligible studies for potential bias using the Cochrane risk-of-bias tool for randomized trials (RoB 1) [29]. This tool evaluates the quality of RCTs across several domains: random sequence generation, allocation concealment, blinding, incomplete outcome data, selective outcome reporting, and other sources of bias. Each domain was rated as “low risk of bias,” “high risk of bias,” or “unclear risk of bias.” The overall risk of bias was determined by considering all domains. The RoB 1 tool was customized in Covidence (Melbourne, Australia) [30]. Any disagreement was resolved independently by a third reviewer (AOW) or through consensus (see Supplementary file, Table 2).

The extracted trial characteristics included: clinical trial registry source (Clinicaltrials.gov, WHO ICTRP, etc.), reporting of study results, indexing of peer-reviewed or study protocol to trial registry, intervention and comparator, allocation concealment, start and end dates of the trial, follow-up time, sample size, study sites (number of sites, geographic location, hospital setting versus other, urban versus rural), recruitment status (active not recruiting, completed, recruiting, suspended, not yet recruiting, or unknown), type of randomization (1:1, 2:1, 3:1, or not reported), blinding (single, double, or not reported), trial phase, and principal investigator (PI) characteristics (sex, affiliation, country). PI sex was determined through information on trial registry source (i.e., Clinicaltrials.gov) and internet searches of PI names.

For RCT protocol characteristics, the following data were extracted: site of patient recruitment (hospitals or clinics, academic institutions, community settings), withdrawal processes (participant withdrawal by choice, administrative withdrawal, study discontinuation), strategies for follow-up of patients (telephone, letter, office, or clinic visits), availability of participant facing materials in other languages, information on barriers to transportation, patient reimbursement or compensation, types of reimbursement or compensation, patient navigation or coaching strategies adopted, information on cultural competency training for clinical research associates or PIs, information on methods for handling missed or late visits, and reasons for excluding patients (missed visits, investigator removal, defaulted clinical follow-up, surgery, death, withdrawal, early termination).

Data were extracted to assess the demographic representativeness of the study, including baseline demographic characteristics (age, race/ethnicity, sex, geographic region) of patients enrolled and those excluded (due to withdrawal, loss to follow-up). Information on the baseline clinical characteristics (intermittent claudication, critical limb ischemia, Rutherford classification, diabetes, hyperlipidemia, hypertension, smoking status, obesity, coronary artery disease, history of congestive heart failure, chronic obstructive pulmonary disease, and other relevant characteristics) of patients enrolled and excluded were also extracted. Furthermore, data on the reporting of demographic characteristics by clinical outcome were extracted, including patency rate/vessel patency, TLR, all-cause mortality/death, amputation (amputation rates, amputation-free survival, minor or major amputations), and serious adverse events/major adverse events. The review assessed the reporting of clinical outcomes by demographic characteristics (age, sex, and race).

Descriptive analysis was used to summarize the features of the trial, publication (e.g., outcomes reported, how analyses were performed), study protocol characteristics, and the reporting of demographic characteristics in the included trials. Meta-regression used the proportion of women enrolled in each study and the mean age of participants in each study as continuous outcomes. Covariates assessed in separate models for each outcome were study year, study location (non-European vs. European), population type (PAD and CLI vs. PAD only), trial length (years), duration of enrollment (months), and the number of study locations. The coefficients represent the difference in outcome (proportion of women or mean age) for a one-unit increment in continuous covariates (study year, trial length, duration of enrollment, or number of study locations), or between locations (non-European vs. European) and population type (PAD and CLI vs. PAD only). The threshold for statistical significance was set at 0.05, meaning that there is a 5% chance of rejecting the null hypothesis when it is true (a type I error). All meta-regression analysis was performed using STATA version 17 (StataCorp LLC, College Station, TX, USA).

## Results

### Search results

**Fig. 1:**
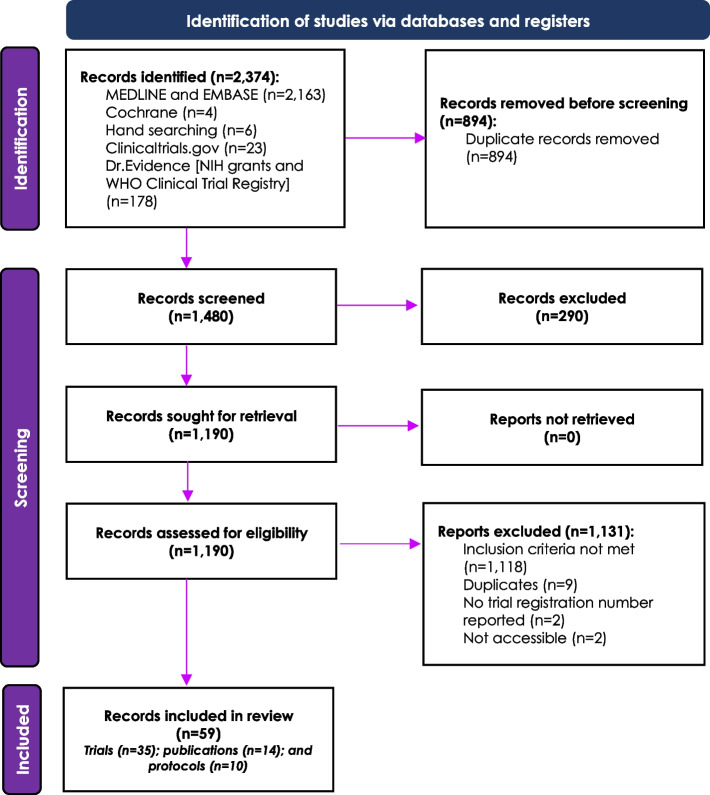
Study Identification Cohort. The number of studies identified via databases and registries, screened, excluded, and included for the final review

Of the 2,374 materials identified, 59 materials (comprising 35 RCTs, 14 publications of RCTs, and 10 protocols) met the inclusion criteria (Fig. 1). All records were unique and reflected different studies.

### Characteristics of RCTs

The 35 RCTs comprised a total of 4,338 trial participants across nine countries (Table 2). The lead PIs were mostly male (31, 89%) and most often affiliated with hospitals (24, 69%), followed by academic institutions (8, 23%). The most common country affiliations of the PIs were Germany (12, 34%), the US (11, 31%), and China (7, 20%). Among the 35 RCTs, 11 (31%) were completed and 6 (17%) reported study results; the remainder were either active, recruiting, not yet recruiting, suspended, or of unknown status. The most common interventions used were drug-coated balloon/drug-eluting balloon (DCB/DEB) (23, 66% of RCTs), followed by drug-eluting stent/drug-coated stent (DES/DCS) (6, 17%), percutaneous transluminal angioplasty (PTA) (3, 8.6%), and bare metal stent (BMS) (3, 8.6%).

**Table 2:** Summary of clinical trials for lower extremity endovascular interventions for the treatment of PAD

**Trial Name and Clinical Trial ID No.**	**Study description**	**Type of randomiz-ation**	**Trial phase**	**Trial blinding**	**Interv-ention model**	**Study location**	**Study sponsor**	**Recruitment status**
EMINENT NCT02921230	The EMINENT study is a prospective, multi-center study confirming the superior effectiveness of the ELUVIA stent versus Self-Expanding Bare Nitinol Stents in the treatment of lesions in the femoropopliteal arteries	2:1 randomiz-ation	N/A	Single (Participant)	Parallel Assign-ment	Geographically spread (Ulsan, Seoul, Pusan, Gyeonggi-do, Jeonju, Bucheon, etc.) Hospital type (University hospitals, hospitals, and VA hospitals)	Boston Scientific Corporation	Active, not recruiting
FIRESTEP NCT04700371	The trial investigates the impact of two different self-expandable nitinol-based stent designs on the target lesion restenosis rate in femoro-popliteal arteries	NR	N/A	None (Open Label)	Parallel Assign-ment	NR- appears single center urban	Kantonsspital Aarau	Not yet recruiting
DCB-SFA NCT02648334	This study evaluates the safety and effectiveness of PTA using DCB for the treatment of SFA/PPA artery in PAD patients	NR	N/A	None (Open Label)	Parallel Assign-ment	Geographically spread (Ulsan, Seoul, Pusan, Gyeonggi-do, Jeonju, Bucheon, etc.) Hospital type (University hospitals, hospitals, and VA hospitals)	Seung-Whan Lee, M.D., Ph.D., Asan Medical Center	Unknown
The PAVENST Trial NCT02212470	To evaluate whether the results of drug eluting balloon are non-inferior to the Nitinol stent implantation in the femoropopliteal segment	NR	Phase 4	Double (Participant, Outcomes Assessor)	Parallel Assign-ment	Urban hospital	Instituto Dante Pazzanese de Cardiologia Medtronic	Completed
ILLUMENATE-BTK NCT03175744	To demonstrate the safety and effectiveness of the Stellarex DCB for the treatment of stenosis or occlusions of BTK arteries	NR	N/A	Single (Participant)	Parallel Assign-ment	Multiple countries, several states, and urban	Spectranetics Corporation Philips Healthcare	Suspended
AcoArt II/BTK China NCT02137577	To determine whether DEB is more effective than common PTA balloon using under in long-term vessel patency and inhibiting restenosis in the infrapopliteal artery	NR	N/A	Single (Outcomes Assessor)	Parallel Assign-ment	Geographically spread in China (Dalian, Beijing Shanghai, Guangzhou, Shenyang, Shijiazhuang, and Tianjin)	Acotec Scientific Co., Ltd	Completed
BIOLUX P-II NCT01867736	To assess the safety and performance of the Passeo-18 Lux Paclitaxel releasing PTA balloon catheter versus the uncoated Passeo 18 PTA balloon catheter for the treatment of stenosis, restenosis or occlusion of the infrapopliteal arteries	1:1 randomiz-ation	N/A	None (Open Label)	Parallel Assign-ment	Hospital and urban mix Geographic (Austria, Belgium [ Bonheiden and Dendermonde], and Germany [ Bad Krozingen, Berlin, and Leipzig]) University and hospital locations	Biotronik AG	Completed
LIMES NCT04772300	This trial evaluates the safety and efficacy of the Magic Touch PTA sirolimus drug-coated balloon in comparison to the treatment with POBA (control device) in patients with infrapopliteal artery disease	1:1 randomiz-ation	N/A	Double (Participant, Outcomes Assessor)	Parallel Assign-ment	Several study locations across Austria and Germany	Jena University Hospital Concept Medical Inc VascuScience GmbH CoreLab Black Forest Center for Clinical Studies, University Hospital Jena	Recruiting
SIRONA NCT04475783	This trial evaluates the safety and efficacy of the Magic Touch PTA sirolimus drug-coated balloon in comparison to the treatment with PTX drug-coated balloon (control device) in patients with femoropopliteal artery disease	1:1 randomiz-ation	N/A	None (Open Label)	Parallel Assign-ment	Multiple sites across Germany	Jena University Hospital Concept Medical Inc Vascuscience CoreLab Black Forest Center for Clinical Studies Jena	Active, not recruiting
SINGA-PACLI NCT02129634	To study the results of DEB-PTA compared to conventional balloon CB-PTA for the treatment of infragenicular lesions in patients with CLI	1:1 randomiz-ation	N/A	Double (Participant, Outcomes Assessor)	Parallel Assign-ment	Urban	Singapore General Hospital Tan Tock Seng Hospital Duke-NUS Graduate Medical School Singapore Clinical Research Institute	Completed
SirPAD NCT04238546	To investigate whether the use of sirolimus-coated balloon catheters in patients with PAD of the femoro-popliteal or BTK segment is not inferior to that of uncoated balloon catheters for major clinical outcomes (unplanned major amputation, target limb re-vascularization)	NR	Phase 3	None (Open Label)	Parallel Assign-ment	Urban hospitals	Nils Kucher	Recruiting
The Chocolate Touch Study NCT02924857	To show sufficient safety and effectiveness of the Chocolate Touchâ„¢ for use in superficial femoral or popliteal arteries with the intention of obtaining regulatory approval to market this device in the United States	1:1 randomiz-ation	N/A	Single (Participant)	Parallel Assign-ment	Urban Several states across the US Mix of University, hospital, and research institutes Multiple countries (US, Austria, Germany, New Zealand)	TriReme Medical, LLC	Active, not recruiting
ILLUMENATE NCT01858428	To evaluate the safety and efficacy of a Paclitaxel-coated PTA catheter in the treatment of patients with PAD	2:1 randomiz-ation	N/A	Single (Participant)	Parallel Assign-ment	Across multiple locations (25) and states in the United States (42) and Austria (2)	Spectranetics Corporation	Completed
NR NCT05415995	To compare the efficacy and safety of DCB (Zylox-Tonbridge) with a similar balloon catheter produced by Acotec	NR	N/A	None (Open Label)	Parallel Assign-ment	Uncertain	Zhejiang Zylox Medical Device Co., Ltd	Recruiting
TIGRIS NCT01576055	To evaluate the safety and effectiveness of the TIGRIS Vascular Stent in the treatment of de novo and restenotic atherosclerotic lesions, a 24 cm in length, in the superficial femoral and proximal popliteal arteries of patients with symptomatic PAD	3:1 randomiz-ation	N/A	None (Open Label)	Parallel Assign-ment	Geographic spread (several states), urban primarily	W.L.Gore & Associates	Completed
SAVAL NCT03551496	To demonstrate a superior patency rate and acceptable safety in below the knee arteries with lesions treated with the DES BTK Vascular Stent System vs. PTA	2:1 randomiz-ation	Phase 3	Single (Outcomes Assessor)	Parallel Assign-ment	Urban—uncertain	Boston Scientific Corporation	Active, not recruiting
HEROES-DCB NCT02812966	Investigators hypothesize in patients presenting with significant PAD with clinical indications for treatment with angioplasty, there will be a difference in 12 month patency between the subjects with Lutonix 035 DCB PTA Catheter and IN.PACT Admiral Paclitaxel-Coated PTA Balloon Catheter	NR	N/A	None (Open Label)	Parallel Assign-ment	Urban and uncertain	Advocate Health Care	Unknown
ILLUMENATE EU NCT01858363	To demonstrate the safety and effectiveness of the CVI Paclitaxel-coated PTA balloon versus bare PTA balloon for the treatment of patients with de novo occluded/stenotic or reoccluded/restenotic lesions of the SFA and popliteal arteries	3:1 randomiz-ation	N/A	Single (Participant)	Parallel Assign-ment	NR	Spectranetics Corporation	Completed
NR NCT02965677	To evaluate the safety and efficacy of the Paclitaxel Releasing Peripheral Balloon Dilatation Catheter (LEGFLOW) compared with the standard balloon (Admiral Xtreme) for the treatment of stenosis or occlusions in femoral popliteal artery	1:1 randomiz-ation	N/A	None (Open Label)	Parallel Assign-ment	Hospitals, urban	ZhuHai Cardionovum Medical Device Co., Ltd	Unknown
Acoart SCB SFA NCT04982367	To compare the efficacy and safety of Sirolimus coated balloon (SCB) versus paclitaxel coated balloon (DCB) in the treatment of femoropopliteal artery stenosis	NR	N/A	None (Open Label)	Parallel Assign-ment	Single urban hospital locatin	Acotec Scientific Co., Ltd	Recruiting
Lutonix BTK Trial NCT01870401	To assess the safety and efficacy of the Lutonix Drug Coated Balloon (DCB) for treatment of stenosis or occlusion of native below-the-knee arteries	2:1 randomiz-ation	N/A	Single (Participant)	Parallel Assign-ment	Urban hospitals primarily	C. R. Bard	Completed
BEST SFA Pilot Study NCT03776799	To compare the efficacy and safety of a stent-avoiding (using DCBs) versus a stent-preferred (using drug eluting or interwoven stents) approach for treatment of complex femoropopliteal lesions TASC II (for the Management of PAD	1:1 randomiz-ation	N/A	None (Open Label)	Parallel Assig-nment	NR	University of Leipzig	Active, not recruiting
COMPARE NCT02701543	To compare two different Paclitaxel coated balloons in the treatment of high grade stenotic or occluded lesions in SFA/PPA artery in PAD patients with Rutherford class 2–4	1:1 randomiz-ation	N/A	None (Open Label)	Parallel Assign-ment	Unsure	University of Leipzig	Active, not recruiting
NR NCT02962232	To evaluate the safety and efficacy of the Paclitaxel Releasing Peripheral Balloon Dilatation catheter compared to the PTA catheter in treatment of stenosis or occlusion in BTK artery	1:1 randomi-zation	N/A	None (Open Label)	Parallel Assign-ment	Unclear—mostly urban	ZhuHai Cardionovum Medical Device Co., Ltd	Unknown
NR NCT03121430	To evaluate the safety and efficacy of drug eluting peripheral vascular stent system for the treatment of SFA stenosis and / or occlusion	1:1 randomiz-ation	N/A	Single (participant)	Parallel Assign-ment	Urban Hospital	Zhejiang Zylox Medical Device Co., Ltd. and Guangzhou Osmunda Medical Device Technology, Inc., Ltd	Unknown
SFA ISR NCT02063672	To assess the safety and efficacy of the Lutonix Drug Coated Balloon for treatment of SFA in-stent restenosis (ISR)	NR	N/A	Single (Outcomes Assessor)	Parallel Assign-ment	Mix of urban and suburban; hospitals, medical centers, academic institutions, and research foundations	C. R. Bard	Completed
SELUTION4SFA Trial NCT05132361	To demonstrate the safety and efficacy of the SELUTION SLR 018 DEB compared to plain (uncoated) balloon angioplasty in the treatment of PAD in the SFA/PPA artery	NR	N/A	Single (Participant)	Parallel Assign-ment	N/A	M.A. Med Alliance S.A. and NAMSA	Not yet recruiting
NR NCT05055297	To demonstrate superior efficacy and equivalent safety of the SELUTION SLR DEB 014 compared to plain (uncoated) balloon angioplasty in the treatment of PAD in the BTK arteries in CLTI patients	NR	N/A	Single (Participant)	Parallel Assign-ment	N/A	M.A. Med Alliance S.A	Recruiting
ZILVERPASS NCT01952457	To evaluate the early and mid-term outcome (after 6 and 12 months) and the long-term (up to 24 months) outcome of the Zilver PTX paclitaxel-eluting stent (Cook) versus bypass surgery for the treatment of TASC C&D femoropopliteal lesions	1:1 randomiz-ation	Phase 4	None (Open Label)	Parallel Assign-ment	Hospital located in different geographic areas in Belgium	Flanders Medical Research Program	Active, not recruiting
BIOPACT-RCT NCT03884257	To investigate the efficacy and safety of stenosis, restenosis or occlusions in the femoropopliteal artery of patients presenting a rutherford classification 2,3 or 4 with a Passeo-18 Lux DCB	1:1 randomiz-ation	N/A	Single (Participant)	Parallel Assign-ment	Unsure—urban hospitals I think	ID3 Medical	Active, not recruiting
RANGER II SFA NCT03064126	To evaluate the safety and effectiveness of the Ranger Paclitaxel Coated Balloon for treating lesions located in the (SFA/PPA) arteries	3:1 randomiz-ation	Phase 3	Single (participant)	Parallel Assign-ment	Community/city hospitals Multiple countries and states Mix of research institutes, Universities, and Hospitals	Boston Scientific Corporation	Active, not recruiting
NR ChiCTR1900023619	To evaluate the efficacy and safety of DCB for treatment of long femoropopliteal Artery disease compared to standard balloon	NR	N/A	NR	Parallel Assign-ment	Single hospital (Tertiary A Hospital)	Beijing Chaoyang Hospital, Capital Medical University	Not yet recruiting
IMPERIAL NCT02574481	To evaluate the safety and effectiveness of Eluvia drug-eluting Vascular Stent System for treating SFA and/or PPA lesions up to 140 mm in length	2:1 randomiz-ation	N/A	Single (Participant)	Parallel Assign-ment	Mix of countries Diverse states across the study locations Mix of community hospitals, academic hospitals, university hospitals, and referral centers	Boston Scientific Corporation	Completed
TRANSCEND NCT03241459	To demonstrate the safety and efficacy of the SurVeil DCB for treatment of subjects with symptomatic PAD due to stenosis of the femoral and/or popliteal arteries	NR	N/A	Single (Participant)	Parallel Assign-ment	Geographic locations US (28 states); Austria (1), Australia (1), Belgium (2), Czechia (2), Germany (4), Italy (1), Latvia (1), and New Zealand (1) Mix of University, research centers, and hospitals	SurModics, Inc	Active, not recruiting
REAL PTX NCT01728441	To compare paclitaxel-eluting stents to paclitaxel-eluting balloons for treating symptomatic PAD of the femoropopliteal artery	NR	N/A	None (Open Label)	Parallel Assign-ment	Urban University (1) Hospital (4) By province: Germany (Leipzig, Hamburg, and Bad Krozingen) Beligum (Bonheiden and Dendermonde)	Provascular GmbH William Cook Europe	Completed

**Trial Name and Clinical Trial ID No.**	**Study start date**	**Study end date**	**Estimated enrollment of participants**	**Actual enrollment of participants**	Sex of PI*	Country of PI*	**Device name of intervention**	**Device name of comparator**
EMINENT NCT02921230	2016	2025	N/A	775	Male	Germany and France	Eluvia Drug-Eluting Vascular Stent System (Boston Scientific)	Innova vascular self-expanding stent system (Boston Scientific)/BMS
FIRESTEP NCT04700371	2022	2024	110	N/A	Male	Switzerland	Name not reported (BMS)	NR (BMS)
DCB-SFA NCT02648334	2016	2021	1080	N/A	Unknown	Republic of Korea	Lutonix DCB	IN.PACT (DCB)
The PAVENST Trial NCT02212470	2014	2019	N/A	85	Male	Brazil	Admiral In.Pact (Medtronic)	Complete SE (Medtronic)/BMS
ILLUMENATE-BTK NCT03175744	2017	2025	354	N/A	Male	USA	Stellarex DCB	Not reported/ PTA balloon catheter
AcoArt II/BTK China NCT02137577	2014	2020	N/A	120	Male	China	Litos/Tulip	Amphirion Deep/PTA balloon catheter
BIOLUX P-II NCT01867736	2012	2014	N/A	72	Male	Germany	Passeo-18 Lux (Biotronik)	Uncoated Passeo-18 PTA balloon catheter
LIMES NCT04772300	2022	2027	230	N/A	Male	Germany	Magic Touch PTA (Concept Medical)	Device name reported/PTA balloon catheter
SIRONA NCT04475783	2021	2027	478	N/A	Male	Germany	IN.PACT Admiral (Medtronic) Luminor (iVascular) Lutonix (BD BARD Peripheral Vascular) Orchid (Acotec Scientific Co., Ltd.) Ranger (Boston Scientific,) SeQuent Please OTW (B. Braun Melsungen AG) Stellarex (Philips)	NR(Commercially available paclitaxel-coated balloon types)
SINGA-PACLI NCT02129634	2013	2018	N/A	136	Male	Singapore	Name not reported (PTA balloon catheter)	Device name not reported/DCB
SirPAD NCT04238546	2020	2028	1,200	N/A	Male	Switzerland	Magic Touch PTA (Concept Medical)	Device name not reported/PTA balloon catheter
The Chocolate Touch Study NCT02924857	2017	2026	585	313	Male	USA and Germany	Chocolate Touch	Lutonix Drug Coated Balloon
ILLUMENATE NCT01858428	2013	2018	N/A	300	Male	USA	EverCross	EverCross 0.035 PTA + Paclitaxel
NR NCT05415995	2022	2024	202	N/A	Unknown	China	Zylox-tonbridge	Acotec
TIGRIS NCT01576055	2012	2017	N/A	267	Male	USA	TIGRIS Vascular Stent (Gore)	Life Stent (Bard)
SAVAL NCT03551496	2018	2029	301	N/A	Male	USA	SAVAL	Device name not reported/ PTA balloon catheter
HEROES-DCB NCT02812966	2016	2019	250	N/A	Male	USA	Lutonix DCB	IN.PACT Admiral Paclitaxel-Coated PTA Balloon Catheter (Medtronic)
ILLUMENATE EU NCT01858363	2012	2020	N/A	294	Male	Germany	CVI Paclitaxel-coated PTA Balloon Catheter	Bare PTA Balloon Catheter
NR NCT02965677	2016	2021	172	N/A	Male	China	LEGFLOW OTW	Admiral Xtreme
Acoart SCB SFA NCT04982367	2021	2024	166	N/A	Male	China	Sirolimus-eluting balloon catheter (Acotec)	Paclitaxel-eluting balloon cathete
Lutonix BTK Trial NCT01870401	2013	2021	N/A	442	Male	USA	Lutonix DCB	Standard uncoated PTA Catheter
BEST SFA Pilot Study NCT03776799	2019	2026	120	N/A	Male	Germany	NR	Device name not reported/DCS
COMPARE NCT02701543	2015	2023	414	N/A	Male	Germany	Ranger DEB (Boston Scientific)	In Pact DEB (Medtronic)
NR NCT02962232	2016	2020	172	N/A	Unknown	China	LEGFLOW OTW	AMPHIRION DEEP
NR NCT03121430	2018	2021	138	N/A	Unknown	China	NR	Cordis Corporation
SFA ISR NCT02063672	2014	2019	N/A	82	Male	USA	Lutonix DCB	Standard Uncoated Balloon Angioplasty Catheter
SELUTION4SFA Trial NCT05132361	2022	2028	300	N/A	Male; Female	Switzerland	SELUTION SLR (MedAlliance)	Uncoated PTA
NR NCT05055297	2022	2028	377	N/A	Male	Germany	SELUTION SLR (MedAlliance)	Plain (Uncoated) Balloon Angioplasty (PTA)
ZILVERPASS NCT01952457	2014	2019	220	N/A	Male	Belgium	Zilver PTX stent (Cook)	Dacron or expanded polytetrafluoroethylene
BIOPACT-RCT NCT03884257	2020	2026	N/A	302	Male	Belgium	Passeo-18 Lux (Biotronik) / PTA balloon catheter	IN.PACT Admiral Paclitaxel-Coated PTA Balloon Catheter (Medtronic)
RANGER II SFA NCT03064126	2017	2023	446	440	Male	USA and Germany	Ranger DEB (Boston Scientific)	Device name not reported/ PTA balloon catheter
NR ChiCTR1900023619	2019	NR	72	36	Male	China	Orchid DCB (Acotec Scientific)	Admiral Xtreme PTA balloon catheter
IMPERIAL NCT02574481	2015	2022	N/A	524	Male	USA and Germany	Eluvia Drug-Eluting Vascular Stent System (Boston Scientific)	Zilver PTX DES
TRANSCEND NCT03241459	2017	2024	446	N/A	Male; Female	USA	Surmodics SurVeil DCB	Medtronic IN.PACT Admiral DCB
REAL PTX NCT01728441	2012	2014	N/A	150	Male	Germany	Zilver PTX stent (Cook) / DES	In.Pact Admiral or In.Pact Pacific (Medtronic) Lutonnix (C.R. Bard)

*BTK* Below-the-knee, *CLTI* Chronic limb-threatening ischemia, *DCB* Drug-coated balloon, *DEB* Drug-eluting balloon, *ISR* In-stent restenosis, *N/A* Not available, *NR* Not reported, *PI* Principal investigator, *PAD* Peripheral artery disease, *PTA* Percutaneous transluminal angioplasty, *PPA* Proximal popliteal artery, *SFA* Superficial femoropopliteal artery, *TASC II* TransAtlantic Inter-Society Consensus

*numbers may not add up due to multiple counts

### Characteristics of RCT protocols

Among the 10 study protocols identified, the majority lacked information relevant to population disparities (Table 3). Four protocols (40%) included information on barriers to transportation, and three (30%) outlined strategies to address these barriers. None of the protocols mentioned patient navigation/coaching strategies, cultural competency training for clinical research associates, or relationship-building/social marketing activities. Seven protocols (70%) discussed follow-up strategies, which included telephone and office/clinic visits. Overall, 7 (70%) of the published protocols planned to recruit patients from hospitals, and 2 (20%) indicated the availability of trial materials in other languages.

**Table 3:** Characteristics of the included clinical trial study protocols for lower extremity endovascular interventions for the treatment of PAD

**Trial Name and Clinical Trial ID No.**	**Protocol accessible**	**Year of protocol publication**	**Method of recruitment**	**Information on barriers to transportation available**	**How transportation barriers were addressed?**	**Patient navigation/ coaching strategies adopted**	**Cultural competency training for clinical research associates**	**Relationship building/social marketing**	**Strategies for follow-up**
EMINENT NCT02921230	Yes	2019	Clinics/Hospitals	Yes	Travel expenses	NR	NR	NR	Telephone; Office /clinic visits
LIMES NCT04772300	Yes	2022	NR	No	NR	NR	NR	NR	NR
SIRONA NCT04475783	Yes	2021	Clinics/Hospitals	No	NR	NR	NR	NR	Telephone; Office /clinic visits; letter
SirPAD NCT04238546	Yes	2022	Other: Academic, Clinics/Hospitals	Yes	NR	NR	NR	NR	Telephone; Office /clinic visits
Lutonix BTK Trial NCT01870401	Yes	2017	NR	No	NR	NR	NR	NR	Telephone; Office /clinic visits
SFA ISR NCT02063672	Yes	2016	Clinics/Hospitals	No	NR	NR	NR	NR	Telephone; Office /clinic visits
BIOPACT-RCT NCT03884257	Yes	2022	NR	No	NR	NR	NR	NR	Unknown
RANGER II SFA NCT03064126	Yes	2018	Clinics/Hospitals	Yes	Stipend	NR	NR	NR	Telephone; Office /clinic visits
IMPERIAL NCT02574481	Yes	2016	Clinics/Hospitals	Yes	Travel expenses	NR	NR	NR	Telephone; Office /clinic visits
TRANSCEND NCT03241459	Yes	2019	Clinics/Hospitals	No	NR	NR	NR	NR	NR

*NR* Not reported

Approximately, 23 (66%) and 7 (20%) of the trials assessed for methodological quality were rated high and low for blinding of participants and personnel. More than half (54%) and 16 (46%) were rated low and unsure regarding allocation concealment (see Supplementary Table 2).

### Characteristics of trial publications

The 14 trial publications comprised a total sample size of 3,964 patients (Table 4). All studies reported age and sex; the overall mean (standard deviation [SD]) age of patients was 68.5 (9.4) years, and two-thirds of patients (67%) were male. Race was provided in 4 of 14 (29%) studies. Among the publications that reported on race/ethnicity (48%), 75% of patients were White, followed by Asian (16%), Black (4.3%), Hispanic (3.0%), other (2.0%), and American Indian/Alaska Native or Native Hawaiian/Pacific Islander (< 1%). None of the publications reported on other demographic characteristics, such as socio-economic status, marital status, or immigration status. Regarding the reporting of treatment effects or outcomes by demographic characteristics, only 4 (29%) publications reported clinical outcomes by sex, age, or race (and 2 did so by sex only); 2 (14%) publications reported primary patency by sex, while one publication reported clinically-driven target lesion revascularization (CD-TLR) by sex.

**Table 4:** Characteristics of included publications, by reporting of demographic characteristics

**Study Characteristics**									**Reporting characteristics: Age**			**Reporting characteristics: Sex**		
**Trial Name and Clinical Trial ID No.**	**Author**	**Country of PI's affiliation**	**Study location(s)**	**Number of study locations**	**Sex of lead author**	**Year of publication**	**Number of enrolled participants**	**Number of excluded participants**	**Reporting of age**	**Age (mean)**	**Age (SD)**	**Reporting of sex**	**Male (n)**	**Female (n)**
EMINENT NCT02921230	Gouëffic et al., 2022 [31]	Germany and France	Austria, Belgium,France, Germany, Ireland,Italy, Netherlands, Spain, Switzerland,UK	60	Male	2022	775	73	Yes	68.9	8.9	Yes	543	232
AcoArt II/BTK China NCT02137577	Jia et al., 2021 [32]	China	China (Dalian, Beijing Shanghai, Guangzhou, Shenyang, Shijiazhuang, and Tianjin)	11	Unknown	2021	120	5	Yes	70.75	8.2	Yes	72	48
BIOLUX P-II NCT01867736	Zeller et al., 2015 [33]	Germany	Austria, Belgium [ Bonheiden and Dendermonde], and Germany [ Bad Krozingen, Berlin, and Leipzig])	6	Male	2015	72	16	Yes	71.25	9.6	Yes	57	15
SINGA-PACLI NCT02129634	Patel et al., 2021 [34]	Singapore	Singapore	2	Male	2021	138	48	Yes	62.5	10	Yes	93	45
The Chocolate Touch Study NCT02924857	Shishehbor et al., 2022 [35]	USA and Germany	US, Austria, Germany, New Zealand	27	Male	2022	333	20	Yes	69.4	9.5	Yes	180	133
ILLUMENATE NCT01858428	Krishnan et al., 2017 [36]	USA	United States and Austria	44	Male	2017	300	30	Yes	69.05	10.05	Yes	176	124
TIGRIS NCT01576055	Laird et al., 2018 [37]	USA	USA	36	Male	2018	267	25	Yes	67.3	9.1	Yes	190	77
ILLUMENATE EU NCT01858363	Schroeder et al., 2017 [38]	Germany	Germany	18	Male	2017	294	54	Yes	68	9	Yes	209	85
COMPARE NCT02701543	Steiner et al., 2020 [39]	Germany	Germany	1	Female	2020	414	22	Yes	68.3	9.65	Yes	260	154
ZILVERPASS NCT01952457	Bosiers et al., 2020 [40]	Belgium	Belgium	5	Male	2020	220	15	Yes	68.6	10.45	Yes	159	61
RANGER II SFA NCT03064126	Sachar et al., 2021 [41]	USA and Germany	USA and Germany	67	Male	2021	376	33	Yes	69.85	9.9	Yes	240	136
NR ChiCTR1900023619	Liao et al., 2022 [42]	China	China	1	Male	2022	60	2	Yes	68.75	8.8	Yes	38	22
IMPERIAL NCT02574481	Gray et al., 2018 [43]	USA and Germany	USA and Germany	68	Male	2018	465	25	Yes	68.15	9.45	Yes	308	157
REAL PTX NCT01728441	Bausback et al., 2019 [44]	Germany	Germany	5	Female	2019	150	28	Yes	68.85	9.55	Yes	102	48

**Study Characteristics**		**Reporting characteristics: Race**									Reporting of other demographic characteristics (socio-economic status, marital status, immigration, etc.)	Clinical outcomes	
**Trial Name and Clinical Trial ID No.**	**Author**	**Reporting of race**	**White (n)**	**Black (n)**	**American Indian/Alaska Native (n)**	**Hispanic/Latino (n)**	**Asian (n)**	**Native Hawaian/Pacific Islander (n)**	**Other (n)**	**Not disclosed (n)**		**Reporting of outcomes by demographics**	**Clinical outcome reported by demographic characteristics**
EMINENT NCT02921230	Gouëffic et al., 2022 [31]	Yes	668	3	1	2	1	0	24	76	No	No	No
AcoArt II/BTK China NCT02137577	Jia et al., 2021 [32]	No	0	0	0	0	0	0	0	0	No	No	No
BIOLUX P-II NCT01867736	Zeller et al., 2015 [33]	No	0	0	0	0	0	0	0	0	No	No	No
SINGA-PACLI NCT02129634	Patel et al., 2021 [34]	No	0	0	0	0	67	0	3	0	No	No	No
The Chocolate Touch Study NCT02924857	Shishehbor et al., 2022 [35]	No	0	0	0	0	0	0	0	0	No	No	No
ILLUMENATE NCT01858428	Krishnan et al., 2017 [36]	No	0	0	0	0	0	0	0	0	No	Sex	Primary patency and CD-TLR
TIGRIS NCT01576055	Laird et al., 2018 [37]	Yes	168	20	0	0	4	0	4	0	No	No	No
ILLUMENATE EU NCT01858363	Schroeder et al., 2017 [38]	No	0	0	0	0	0	0	0	0	No	Sex	Primary patency
COMPARE NCT02701543	Steiner et al., 2020 [39]	No	0	0	0	0	0	0	0	0	No	No	No
ZILVERPASS NCT01952457	Bosiers et al., 2020 [40]	No	0	0	0	0	0	0	0	0	No	No	No
RANGER II SFA NCT03064126	Sachar et al., 2021 [41]	Yes	214	24	1	29	102	0	1	5	No	Yes	No
NR ChiCTR1900023619	Liao et al., 2022 [42]	No	0	0	0	0	0	0	0	0	No	No	No
IMPERIAL NCT02574481	Gray et al., 2018 [43]	Yes	313	32	4	24	113	1	4	2	No	No	No
REAL PTX NCT01728441	Bausback et al., 2019 [44]	No	0	0	0	0	0	0	0	0	No	No	No

*NR* Not reported, *PI* Principal investigator, *SD* Standard deviation

### Meta-regression by demographic characteristics

**Fig. 2:**
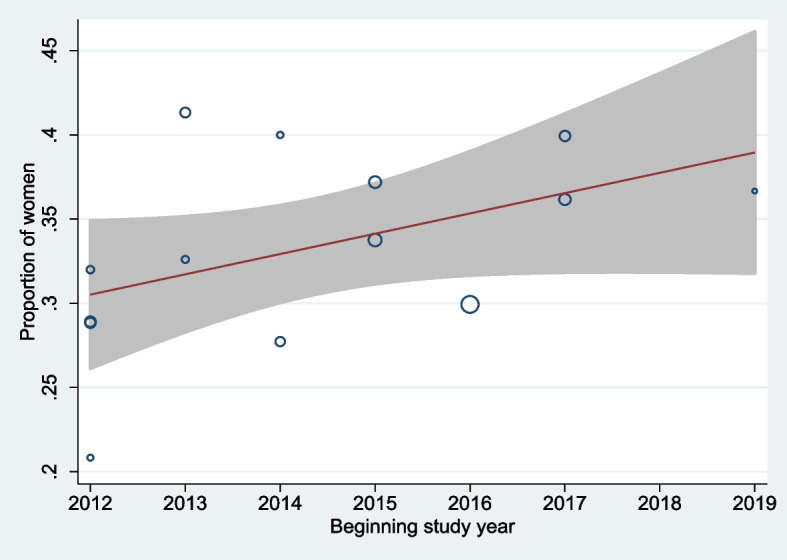
Meta-Analysis Bubble Plot of the Proportion of Women Enrolled by Study Start Year. The bubbles are drawn with sizes proportional to the contribution of individual studies towards the linear prediction

Across all 14 trial publications, women were underrepresented, accounting for 33% of participants. The meta-regression analysis revealed that 5.9% more women were enrolled in non-European trials (36%) than in European trials (30%). However, meta-regression analysis shows the proportion of women enrolled in the trials increased over time, a finding that was not statistically significant (Table 5). While the proportion of women enrolled varied by study population type, trial length, enrollment duration, or the number of study locations, a significantly higher proportion of women were enrolled in studies in non-European countries (US, China, Singapore, New Zealand) compared to European countries (Table 5). Figure 2 shows the proportion of women increased between 2012 and 2019 (reflected by the trial start year); however, this finding is non-significant.

**Table 5:** Meta-regression results

**Outcome/Covariate**	**Coefficient**	***p*-value**
Proportion of Women		
Study year	0.012	0.096
Study location (Europe vs non-Europe)	0.059	0.032
Population type (PAD and CLI vs PAD only)	0.013	0.77
Trial length (years)	0.0086	0.25
Duration of enrollment (months)	0.0003	0.84
Number of study locations	0.0003	0.63
Mean Age of Participants		
Study year	0.187	0.46
Study location (Europe vs non-Europe)	-0.647	0.55
Population type (PAD and CLI vs PAD only)	0.193	0.90
Trial length (years)	-0.035	0.90
Duration of enrollment (months)	-0.042	0.29
Number of study locations	0.013	0.57

*CLI* Critical limb ischemia, *PAD* Peripheral artery disease

The mean age of participants did not significantly differ by study year, location, study population type, trial length, duration of enrollment, or the number of study locations (Table 5).

## Discussion

Previous studies have emphasized the poor representation of women and racially/ethnically diverse or underrepresented minorities (URMs) in cardiovascular trials [22, 45, 46]. Efforts have been made to address this disparity by implementing innovative trial designs that prioritize diverse enrollment recruitment processes and minimize sex-specific exclusion criteria [46]. For instance, the ELEGANCE registry, a global clinical peripheral vascular disease (PVD) registry, was specifically designed to enroll diverse patient populations that have been historically underrepresented in PVD trials [47]. As of December 2022, the registry achieved an enrollment of 44% women and 47% URMs in the US [47]. This registry’s focus on diverse enrollment is crucial for enhancing the generalizability of study findings and providing optimal individualized care for all patients with PAD.

This analysis revealed a limited representation of female physicians participating as PIs in clinical trials. Previous studies have shown that race concordance between patients and providers can lead to better patient-clinician relationships, better disease management, and improved outcomes [48–50]. This suggests that increasing the diversity of PIs and study teams could impact the level of comfort and trust of the diverse patients these studies aim to recruit. To increase diversity in clinical research teams, it is imperative to invest in equity initiatives that prioritize promoting demographic representativeness among physicians and fostering diverse participation in clinical research globally (and more specifically, RCTs) [47, 51]. If successful, such initiatives would improve patient-physician concordance and help to enhance the diversity of clinical trial participants, improving the validity and relevance of research findings.

This review supports previous findings that demonstrate a lack of reporting and representation of participant sub-groups beyond age, sex, and race. Information pertaining to income, education, language proficiency, immigrant status, or other relevant characteristics were absent in the published RCTs [24, 46, 52–55]. The absence of such information hinders our ability to generalize treatment outcomes to specific sub-groups and understand the potential moderating effects of these factors [56, 57]. Representation of diverse sub-groups is crucial as it promotes inclusivity and ensures comprehensive reporting in clinical trials, enabling the application of trial findings to diverse populations and informing equitable healthcare practices. To encourage consistency in how such results are reported, some journals, such as those published by the American Heart Association, provide guidance for authors submitting manuscripts that report health differences by race/ethnicity [58].

Insufficient attention has been given to addressing the geographic and regional variability in PAD RCTs. This variability is likely influenced by local policies that can significantly impact the conducting and reporting of clinical trials. Regional policies, including regulatory requirements, reimbursement practices, and research infrastructure requirements, can create barriers and affect the feasibility of conducting and reporting trial data. Such policies may introduce increased costs or burdens that hinder participation or data collection, ultimately impacting the generalizability of treatment outcomes. It is crucial to acknowledge and account for these regional policy differences to ensure the validity and applicability of trial findings across diverse geographical settings.

In the identified protocols, there was a reliance on traditional recruitment strategies that primarily targeted participants from clinics and academic settings. Careful site selection can help increase the diversity of both patient populations and the research team. Additionally, it is important to recognize the need for broader inclusion and adoption of non-traditional recruitment strategies to enhance the representation of diverse and URMs in clinical trials. Thus, movement toward the inclusion and adoption of non-traditional recruitment strategies are necessary to boost the inclusion of diverse and under-represented groups. Expanding the eligibility criteria beyond traditional parameters, providing training on implicit bias and cultural competence, and increasing the diversity of funding committees and reviewers may help increase diversity in trials [46].

The effective management of PAD requires a multifaceted approach with strategies anchored by several factors, such as patients, healthcare systems and providers, and scientific advancements. To address the complexities associated with PAD, it is important for trial protocols to integrate approaches that address each of these components [59]. Collaborative initiatives among various stakeholders (academia, regulatory bodies, industry stakeholders, and healthcare payors) are crucial in facilitating the conduct of clinical trials focused on cardiovascular conditions, including PAD [60]. Such inter-agency collaborations foster the timely introduction of innovative therapies and enhance the overall management of cardiovascular diseases.

A major strength of this study is the inclusion of research from around the globe, rather than just a single country or geographic location, which increases the external validity of the findings. This was accomplished by using a variety of databases from different sources, thereby maximizing the inclusion of published trials and increasing the volume of included studies that evaluated the diversity of clinical trials. Additionally, this study used three types of data sources that centered on RCTs (trial registrations, protocols, and peer-reviewed publications), which offer stakeholders comprehensive information about the diversity of clinical trials from trial design, trial reporting, and trial outcomes on studies that are in progress or have been completed. The findings offer insights to inform policy and clinical decision-making in RCTs.

This study has several limitations. The study was limited by the small number of studies identified, which potentially threatens the generalizability of the study findings. It may be that expanding the search criteria would include more studies; the requirement of a sample size of at least 50 participants may have excluded studies with more diverse patient populations. This study observed missing data or inconsistencies between the reporting of information in clinical trial registries versus publications. Other studies have reported on the quality of clinical trial data submission and indicated a need to improve the reporting of results posted in trial registries [61]. For instance, in Clinicaltrials.gov, some studies reported extensive details regarding locations, patient population, included protocols, and results, while other studies reported limited information on trial features. Unless additional details are provided in the publications, the variability in the quality of reporting is a limitation. The use of non-study level variables (proportion of women, mean age) in a meta-regression should be interpreted with caution since they are subject to ecological fallacy [62]. Lastly, the variability in the methodological quality ratings (blinding, allocation concealment, etc.), could potentially introduce a source of bias in the study results, impacting the reliability of the conclusions drawn from this analysis.

Despite these limitations, this review holds implications for clinical practice, policy, and future research. First, these findings highlight potential issues that can undermine the reliability and validity of study findings in lower-extremity PAD RCTs. Addressing these issues is crucial to enhancing the evidence-base for clinical decision-making and improving clinical outcomes for the management of PAD. Additionally, the observed inequities in clinical trial study populations emphasize the importance of health equity for URMs. Regulatory and decision-making bodies globally have promoted guidelines aimed at improving representation in clinical trials [63–65]. Countries and regions without universally-accepted guidelines promoting clinical diversity should pursue the development of such guidelines, using existing resources as guides. In the US, the Food and Drug Administration recently released the final guidance on Clinical Trial Diversity Plans [66] driven by legislative mandates. Approaches for inclusive trials have been reported in the literature [47, 67–71]. Standardization efforts are needed to ensure transparency, accountability, and progress in achieving health equity while considering the cultural and social context of trial locations.

Future research must encourage investigators and life sciences industry representatives to increase investments and diversify resources to improve the design of clinical research. This includes expanding the inclusion of regions and populations underrepresented in clinical trials. Integrating a health equity lens into trial design is crucial, with a focus on ensuring fair and equitable representation of diverse populations. It is equally important to emphasize the reporting and the interpretation of trial results by key clinical outcomes through an equity perspective [72]. In addition to addressing representation, it is essential to consider the potential burden and costs that participants may incur when participating in clinical trials. Direct costs (e.g., travel expenses to the trial site) and indirect costs (e.g., productivity loss) can have an impact on participant motivation and retention. Thus, PIs should explore existing incentives (e.g., travel reimbursement) and develop strategies to boost retention in clinical trials [73]. Future research should consider exploring the role of demographic characteristics beyond age, sex, and race in treatment outcomes.

### Supplementary Information

**Additional file 1: Table S1. **Search strategy. **Table S2.** Quality assessment of the included studies.

## Data Availability

Data supporting the findings of this study are available from the corresponding author (AOW), upon reasonable request.

## Abbreviations

BMS: Bare metal stent
DCB: Drug-coated balloon
DCS: Drug-coated stent
DEB: Drug-eluting balloon
DES: Drug-eluting stent
FPA: Femoropopliteal artery
MALE: Major adverse limb event
PAD: Peripheral artery disease
PICOS: Population, interventions, comparators, outcomes, and setting
PRISMA: Preferred Reporting Items for Systematic Reviews and Meta-Analyses
PTA: Percutaneous transluminal angioplasty
PVD: Peripheral vascular disease
RCT: Randomized controlled trial
SD: Standard deviation
SFA: Superficial femoral artery
TLR: Target lesion revascularization
URM: Underrepresented minority
