# Supplementary material for: Diversity in randomized clinical trials for peripheral artery disease: a systematic review
Source: Int J Equity Health. 2024 Feb 13;23:29. doi: 10.1186/s12939-024-02104-8 (PMC10865563; doi:10.1186/s12939-024-02104-8)
Supplement: Supplementary file 1 — Additional file 1: Table S1. Search strategy. Table S2. Quality assessment of the included studies. [file 12939_2024_2104_MOESM1_ESM.docx]

**Additional File 1: Search Strategy**

Table 1. Search Strategy

| **PICO Strategy** | **Search Terms** |
| --- | --- |
| Population | SFA lesions OR FPA lesions OR Severe Limb Ischemia OR popliteal lesions OR tibial lesions OR Peripheral arterial disease OR Peripheral artery disease PAD OR critical limb ischemia OR intermittent claudication OR critical limb OR limb ischem OR claudication OR chronic limb-threatening ischemia OR limb ischemia or limb threat* OR ischaemia AND (leg OR legs OR limb OR limbs) |
| Interventions | Peripheral interventions OR stents OR drug eluting stents OR drug coated stents OR drug coated balloons OR drug-coated balloons OR balloon angioplasty OR plain balloon OR bare metal stent OR paclitaxel eluting stent OR paclitaxel-coated balloon OR PTA OR percutaneous transluminal angioplasty OR uncoated PTA OR lower extremity OR endovascular procedures OR angioplasty, balloon |
| Comparators | Endovascular intervention OR bare metal stent OR BMS OR drug-coated balloon OR DCB OR drug-eluting balloon OR DEB plain old balloon angioplasty OR POBA percutaneous transluminal angioplasty OR PTA OR drug-coated stent OR DCS OR drug-eluting stent OR DES |
| Outcomes | Patency rate OR vessel patency OR target lesion revascularization OR TLR OR all-cause mortality OR all-cause death OR amputation OR amputation rates OR amputation-free survival OR minor amputation OR major amputation OR serious adverse event OR major adverse event OR wound healing |

Source: Long et al. (2023) Diversity in Clinical Trial Inclusion for Peripheral Artery Disease (PAD) Lower Extremity Endovascular Interventions: A Systematic Review Protocol^25^

Table 2. Quality Assessment of the Included Studies

| **Trial Name and Clinical trial ID No.** | **Sequence generation** | **Allocation concealment** | **Blinding of participants and personnel** | **Blinding of outcome assessment** | **Incomplete outcome data** | **Selective reporting** | **Other sources of bias** |
| --- | --- | --- | --- | --- | --- | --- | --- |
| EMINENT (NCT02921230) | Low | Low | Low | Low | Low | Low | Low |
| FIRESTEP (NCT04700371) | Unsure | Unsure | High | High | Unsure | Unsure | High |
| DCB-SFA (NCT02648334) | Unsure | Unsure | High | High | Unsure | High | High |
| The PAVENST Trial (NCT02212470) | Unsure | Unsure | Low | Low | Unsure | Unsure | Unsure |
| ILLUMENATE-BTK (NCT03175744) | Low | Low | High | High | Unsure | Unsure | High |
| AcoArt II/BTK China (NCT02137577) | Low | Unsure | High | Low | Low | Unsure | High |
| BIOLUX P-II (NCT01867736) | Low | Low | High | Low | Low | Low | High |
| LIMES (NCT04772300) | Low | Unsure | Low | Low | Unsure | Unsure | Low |
| SIRONA (NCT04475783) | Low | Low | High | Low | Unsure | Unsure | Unsure |
| SINGA-PACLI (NCT02129634) | Low | Low | Low | Low | Low | Low | Low |
| SirPAD (NCT04238546) | Low | Low | High | High | Unsure | Unsure | Unsure |
| The Chocolate Touch Study (NCT02924857) | Low | Low | High | Low | Unsure | Low | Unsure |
| ILLUMENATE (NCT01858428) | Low | Low | High | Low | Low | Low | High |
| NR (NCT05415995) | Low | Unsure | High | Unsure | Unsure | Unsure | Unsure |
| TIGRIS (NCT01576055) | Low | Low | High | High | High | High | High |
| SAVAL (NCT03551496) | Low | Unsure | Unsure | Low | Unsure | Unsure | Unsure |
| HEROES-DCB (NCT02812966) | Low | Unsure | High | Unsure | Unsure | Unsure | Unsure |
| ILLUMENATE EU (NCT01858363) | Low | Low | High | Low | High | Low | Unsure |
| NR (NCT02965677) | Low | Unsure | High | Unsure | Unsure | Unsure | Unsure |
| Acoart SCB SFA (NCT04982367) | Unsure | Unsure | High | Unsure | Unsure | Unsure | Unsure |
| Lutonix BTK Trial (NCT01870401) | Low | Low | High | Low | Unsure | Unsure | Unsure |
| BEST SFA Pilot Study (NCT03776799) | Low | Unsure | High | High | Unsure | High | High |
| COMPARE (NCT02701543) | Low | Low | High | Low | Low | Low | Unsure |
| NR (NCT02962232) | Low | Unsure | High | Unsure | Unsure | Unsure | Unsure |
| NR (NCT03121430) | Low | Unsure | Unsure | Unsure | Unsure | Unsure | Unsure |
| SFA ISR (NCT02063672) | Low | Low | Low | Low | Low | Low | Unsure |
| SELUTION4SFA Trial (NCT05132361) | Low | Unsure | Unsure | Unsure | Unsure | Unsure | Unsure |
| NR (NCT05055297) | Low | Unsure | Unsure | Unsure | Unsure | Unsure | Unsure |
| ZILVERPASS (NCT01952457) | Low | Low | High | High | Low | Unsure | Low |
| BIOPACT-RCT (NCT03884257) | Low | Unsure | Unsure | Unsure | Unsure | Unsure | Unsure |
| RANGER II SFA (NCT03064126) | Low | Low | Low | Low | Low | Low | High |
| NR (ChiCTR1900023619) | Low | Low | High | Low | Low | High | Low |
| IMPERIAL (NCT02574481) | Low | Low | Low | Low | Low | Low | Low |
| TRANSCEND (NCT03241459) | Low | Low | High | Low | Unsure | Unsure | Low |
| REAL PTX (NCT01728441) | Low | Low | High | High | Low | Low | High |

NR, not reported
